# Consensus on Hearing Aid Candidature and Fitting for Mild Hearing Loss, With and Without Tinnitus: Delphi Review

**DOI:** 10.1097/AUD.0000000000000140

**Published:** 2015-06-24

**Authors:** Magdalena Sereda, Derek J. Hoare, Richard Nicholson, Sandra Smith, Deborah A. Hall

**Affiliations:** 1 National Institute for Health Research Nottingham Hearing Biomedical Research Unit, Nottingham, United Kingdom; 2 Otology and Hearing Group, Division of Clinical Neuroscience, School of Medicine, University of Nottingham, Nottingham, United Kingdom; and 3 Nottingham Audiology Services, Nottingham University Hospitals National Institutes of Health Trust, Nottingham, United Kingdom.

**Keywords:** Delphi review, Hearing aids, Mild hearing loss, Tinnitus

## Abstract

**Objectives:**

In many countries including the United Kingdom, hearing aids are a first line of audiologic intervention for many people with tinnitus and aidable hearing loss. Nevertheless, there is a lack of high quality evidence to support that they are of benefit for tinnitus, and wide variability in their use in clinical practice especially for people with mild hearing loss. The aim of this study was to identify a consensus among a sample of UK clinicians on the criteria for hearing aid candidature and clinical practice in fitting hearing aids specifically for mild hearing loss with and without tinnitus. This will allow professionals to establish clinical benchmarks and to gauge their practice with that used elsewhere.

**Design:**

The Delphi technique, a systematic methodology that seeks consensus amongst experts through consultation using a series of iterative questionnaires, was used. A three-round Delphi survey explored clinical consensus among a panel of 29 UK hearing professionals. The authors measured panel agreement on 115 statements covering: (i) general factors affecting the decision to fit hearing aids, (ii) protocol-driven factors affecting the decision to fit hearing aids, (iii) general practice, and (iv) clinical observations. Consensus was defined as a priori ≥70% agreement across the panel.

**Results:**

Consensus was reached for 58 of the 115 statements. The broad areas of consensus were around factors important to consider when fitting hearing aids; hearing aid technology/features offered; and important clinical assessment to verify hearing aid fit (agreement of 70% or more). For patients with mild hearing loss, the greatest priority was given by clinicians to patient-centered criteria for fitting hearing aids: hearing difficulties, motivation to wear hearing aids, and impact of hearing loss on quality of life (chosen as top five by at least 64% of panelists). Objective measures were given a lower priority: degree of hearing loss and shape of the audiogram (chosen as top five by less than half of panelists). Areas where consensus was not reached were related to the use of questionnaires to predict and verify hearing aid benefit for both hearing and tinnitus; audiometric criteria for fitting hearing aids; and safety of using loud sounds when verifying hearing aid fitting when the patient has tinnitus (agreement of <70%).

**Conclusions:**

The authors identified practices that are considered important when recommending or fitting hearing aid for a patient with tinnitus. More importantly perhaps, they identified practical issues where there are divided opinions. Their findings inform the design of clinical trials and open up debate on the potential impact of practice differences on patient outcomes.

## Abstract 2

This study identified areas of consensus among 29 United Kingdom hearing professionals on the criteria for hearing aid candidature and clinical practice in fitting devices for mild hearing loss with and without tinnitus. Consensus was strong for factors important to consider when fitting hearing aids, hearing aid technology/features offered, and important clinical assessment to verify hearing aid fit. The results will inform the design of clinical trials and open up debate on the potential impact of practice differences on patient outcomes.

## INTRODUCTION

Tinnitus is a major problem affecting 10 to 15% of adults depending on the study (Hoffman & Reed 2004), with about 20% of those experiencing symptoms that negatively affect the quality of life and require clinical intervention (Davis & El Rafaie 2000). Some of the most well-known management strategies for tinnitus are education and reassurance, relaxation, cognitive behavioral therapy (CBT), psychological counseling, some form of sound therapy including hearing aids or sound generators, or a combination of these approaches (Hoare et al. 2012). There is no standard procedure for the diagnosis and management of tinnitus, and the approach taken depends strongly on the professional background of the clinician (medic, audiologist/hearing therapist, clinical psychologist), as well as country-specific guidelines or lack thereof (Henry et al. 2008; Department of Health 2009; Biesinger et al. 2011; Hall et al. 2011; Hoare & Hall 2011; Cima et al. 2012; Tunkel et al. 2014).

In the United Kingdom, audiology is the main provider of care for people with tinnitus (Hall et al. 2011) and the most common audiologic management strategy is education and reassurance, combined with sound therapy (e.g., hearing aids, Department of Health 2009; Hoare et al. 2012). Hearing aids are primarily prescribed to overcome hearing loss, which is often associated with tinnitus (Coles 1995; Dobie 2004). They also reduce listening effort, improve communication, and so can reduce stress and anxiety that may be associated with hearing loss and are common to tinnitus (Surr et al. 1985; Carmen & Uram 2002). However, hearing aids may also be beneficial for tinnitus by amplifying environmental sound, thereby masking or providing distraction from tinnitus. Some authors postulate that hearing aids may have a physiological effect on tinnitus-related brain activity, by recalibrating central gain (Schaette & Kempter 2006; Schaette et al. 2010) or preventing maladaptive neural plasticity triggered by the damage to the auditory periphery and inducing “secondary” plasticity in the auditory system (Willott 1996; Noreña 2011).

UK (Department of Health 2007; British Society of Audiology (BSA) Practice Guidance 2012) and US (American Academy of Audiology (AAA) Task Force 2006; Tunkel et al. 2014) guidelines share many common recommendations for the audiologic management of adults with hearing impairment, where hearing aids play a major role. The referenced guidelines are mostly relevant to the management of sensorineural hearing loss, irrespective of severity, that is, there are no specific recommendations for patients with mild sensorineural hearing loss. Little attention is paid to the management of comorbid symptoms such as tinnitus. The BSA Practice Guidance (2012) emphasizes assessing individual needs and lists tinnitus and client’s attitude toward it as one of the factors important to assess to inform the best management strategy but without recommending specific treatment options. The AAA Task Force document (2006) puts tinnitus in the list of nonauditory needs of the patient that may interfere with auditory needs to determine success with amplification. None of the above documents contain any guidelines about the management of patients with hearing loss and tinnitus. Furthermore, despite the extensive information available about hearing aid technology and programming features, there are no recommendations as to which features might be useful for patients with tinnitus (Tunkel et al. 2014). Although the guidelines for the general management of hearing impairment in both BSA Practice Guidance (2012) and AAA Task Force document (2006) contain similar elements, the same is not the case for tinnitus.

There have been a number of tinnitus-specific management guidelines. Few of them provide specific recommendations of best practice (Department of Health Good Practice Guide 2009; Tinnitus Research Initiative [TRI] algorithm, Biesinger et al. 2011, but see Tunkel et al. 2014). This leaves a lot of scope for different interpretations of the guidelines for individual patients. As well as differences, there are some common elements to most standard and nonstandard treatment pathways such as similar protocols for diagnostic assessment and classification of tinnitus severity, as well as similar management options (Baguley et al. 2013). Among those treatment pathway elements, addressing hearing difficulties is one of the care components, with hearing aids being one common management option (Searchfield et al. 2010; Henry et al. 2008; Department of Health Good Practice Guide 2009; Biesinger et al. 2011; Cima et al. 2012).

Variability in clinical practice has developed from differences in experience and opinion rather than from different practice guidelines. There is mixed evidence to support clinical efficacy and effectiveness of hearing aids for tinnitus. A recent Cochrane review by Hoare et al. (2014) evaluating amplification with hearing aids for tinnitus concluded that there is a lack of high quality evidence. The review reported the results of one randomized controlled trial (RCT; Parazzini et al. 2011), which demonstrated that there was no difference in improvement in terms of reduced self-reported tinnitus severity and tinnitus loudness between participants prescribed hearing aids (with normal low-frequency hearing but high-frequency hearing loss) and participants prescribed a sound generator devices. There is lower level evidence for the effects of hearing aids in the form of non-RCTs, as described by the scoping review by Shekhawat et al. (2013). In general, those studies support the use of hearing aids for tinnitus management. These nonrandomized studies however provide an estimate effect that is less reliable. Given such a lack of research to support the delivery of evidence-based practice, further research is warranted (Tunkel et al. 2014).

Reflective of the lack of high level evidence to guide practice, there are no uniform criteria for candidacy and no standard fitting procedure when it comes to providing hearing aids to primarily address a complaint of tinnitus, for example, where there is no complaint of a hearing handicap or only a mild hearing loss. Our survey of tinnitus management in audiology departments across England found that 47% of hearing healthcare professionals used different criteria when deciding on candidature for a hearing aid for patients with hearing loss and tinnitus compared with patients with a hearing loss alone (Hoare et al. 2012). The most common difference was a greater likelihood to prescribe a hearing aid in cases of mild sensorineural hearing loss when the patient also complains of tinnitus. This difference is interesting as many authors put emphasis on the importance of assessing and addressing hearing loss/difficulties as a step in the management of tinnitus, but there are no previous reports of using different criteria for candidature for hearing aids between patients with and without tinnitus. For candidates, recommended fitting options vary in the literature. The main variability surrounds the following issues: (i) whether to always fit bilaterally (Del Bo & Ambrosetti 2007; Trotter & Donaldson 2008); (ii) the relationship between the range of amplification and tinnitus pitch, with some authors stating that amplification would be effective for tinnitus only if tinnitus pitch is within the amplified frequency range (Moffat et al. 2009; Schaette et al. 2010); (iii) which prescription formulae to use (Shekhawat et al. 2013); and (iv) what other forms of therapy should be prescribed together with hearing aids (e.g., counseling or combination hearing aids; Hobson et al. 2010; Searchfield et al. 2010; Baguley et al. 2013).

The use of hearing aids in tinnitus management will in many cases be associated with an improvement in hearing handicap and associated quality of life and that complicates the interpretation of how much hearing aids specifically affect tinnitus handicap (Hobson et al. 2010). Patients with mild hearing losses (pure-tone average between 20 and 40 dB HL) would be predicted to experience the smallest improvement in hearing handicap, so a study of this group could be illuminating. To be able to investigate such effect, first one needs to define an assessment and fitting protocol that reasonably reflects current clinical practice. However, patients with mild hearing losses are the group for whom current clinical practice varies the most (Parazzini et al. 2011; Hoare et al., 2012). We were interested to know therefore how this variability manifested in clinical practice in the United Kingdom. Our question concerned what options are most consistent and most diverse when it comes to deciding on candidature and hearing aid fitting procedures for patients with mild hearing loss when patients do or do not have bothersome tinnitus. To do this, we used the Delphi survey methodology that is widely used to forecast or ascertain consensus on issues, such as best clinical practice, research priorities, and service planning (Powell 2003; Avery et al. 2005). The Delphi survey is a systematic methodology that uses a sequential set of questionnaires with controlled feedback, in an iterative and interactive manner (Lindstone & Turoff 1975). The steps and decision methods are predefined, setting it apart from other less formalized consensus approaches. The use of Delphi technique is well justified in this case because there is an incomplete state of knowledge (Powell 2003). A major strength of the Delphi methodology is that people taking part bring a wide range of expertise or experience to the decision-making process. It also gathers the opinions in a relatively time efficient and cost-effective way. One of the challenges however is the choice of appropriate panel. Careful consideration is needed of the inclusion criteria according to qualifications, credibility, and willingness to participate so the panel represents a range of experiences relevant to the question being addressed (Powell 2003). Another issue is defining the level of consensus. Studies that aim to define treatment protocols and medical procedures (Hill et al. 2012; Olthof et al. 2013; Vitale et al. 2013; Westby et al. 2013) err toward higher predefined levels of agreement to represent consensus (usually ≥80 to 90% agreement). Studies where the outcome is less critical to immediate health or life (e.g., to define research priorities) can afford a less stringent interpretation of consensus (Lindeman 1975; Bond & Bond 1982).

In summary, our aim was to identify, according to clinical opinion, which elements of practice are most commonly agreed as important in determining whether someone with mild hearing loss, with or without tinnitus, should be offered a hearing aid and how the hearing aid might be fitted. Although the study was confined to the UK model of audiology services, the results are informative for other countries.

## MATERIALS AND METHODS

### Design

The study was managed by academics at the National Institute for Health Research Nottingham Hearing Biomedical Research Unit. Clinicians from Nottingham Audiology Services were involved in the survey design.

We predefined consensus as ≥70% agreement (Agree or Strongly Agree) among the panel. The primary aim of our study was to identify clinical consensus on the criteria for hearing aid candidature and practices for fitting hearing aids for mild hearing loss with and without tinnitus to inform the design of clinical trials. We did not wish to draw any conclusions about the effectiveness of current practice or formulate any clinical recommendations or guidelines at this stage. Therefore, a consensus of ≥70% agreement was considered appropriate to interpret the data. In addition, Kendall’s coefficient of concordance W was calculated to assess the degree of agreement between panelists in the final round of the questionnaire (round 3). Statistical analysis was performed in R 3.0.2 (R Core Team 2013). Multiple imputation was performed for missing data (van Buuren 2012).

**Fig. 1.**
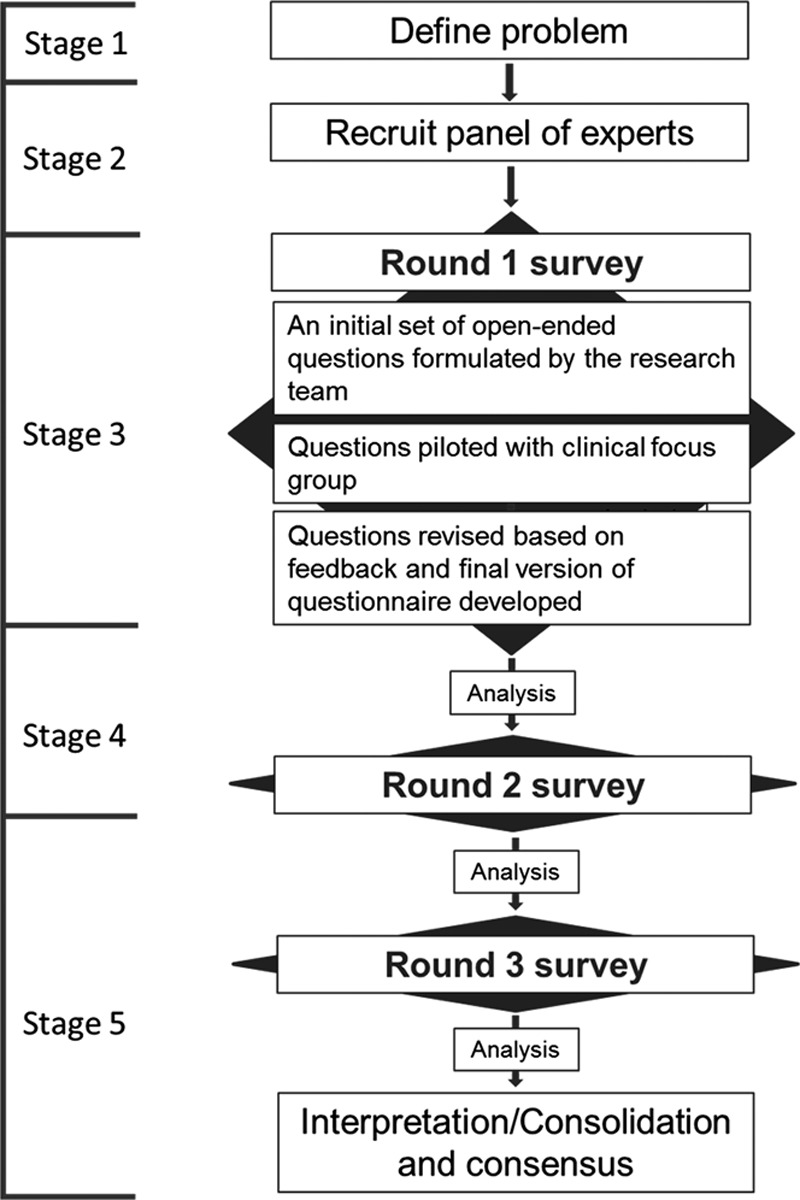
Delphi review process.

The study design followed a standard Delphi survey methodology (Lindstone & Turoff 1975) where three rounds of questionnaires were distributed among the expert panel (Fig. 1). The printed version of the round 1 questionnaire was posted to the panel for return in a prepaid envelope. Feedback indicated that most of our panelists would prefer electronic questionnaires so these were provided in further rounds. In each round, participants were requested to complete and return the questionnaire within 3 weeks. Reminders were sent 1 week before the deadline. Questionnaire responses were anonymized by associating a random number with each panel member. Researchers performing the analysis were blind as to which questionnaire was completed by which expert.

Our Delphi protocol consisted of five stages.

### Stage 1: Defining the Problem

Our long-term goal is to design and conduct a gold standard RCT to evaluate the efficacy of hearing aid interventions for people with tinnitus. A key step in this process is to document and better understand current practices for fitting hearing aids to ascertain the feasibility of conducting such a study. The immediate goal of the current study was to identify consensus to establish clinical benchmarks and to enable clinical professionals to gauge their practice with that elsewhere.

### Stage 2: Recruitment of the Expert Panel

An invitation letter was sent to 65 UK-based audiology and hearing therapy staff who had responded to a previous survey of tinnitus management (Hoare et al. 2012). The letter contained a description of the project goals, the Delphi process, and the timeline. This survey targeted only those audiology departments offering a tinnitus service. We further invited clinicians working in seven audiology centers actively collaborating with Nottingham Hearing Biomedical Research Unit on other research projects. The study was advertised at the British Tinnitus Association (BTA) 2012 conference and in the BTA’s Quiet magazine. Hearing professionals were eligible to participate if they had (1) experience of assessing and fitting hearing aids to patients with and without tinnitus, (2) interest in the topic of fitting hearing aids for mild hearing losses, and (3) willingness to share their opinions. We did not seek to further restrict the criteria for participation in the survey. Panel members did not receive any financial compensation for participating, however, to encourage response, a prize draw was open to all participants completing the study.

**Fig. 2.**
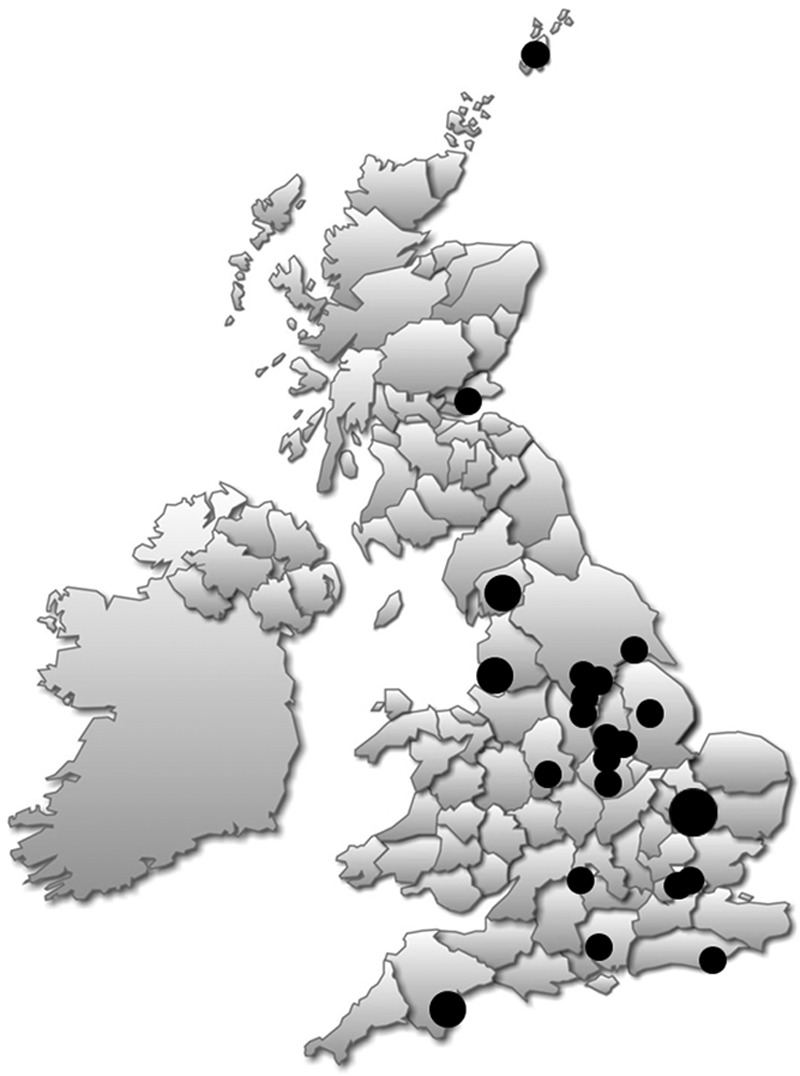
Map showing the centers in the United Kingdom that took part in the Delphi review.

Forty-two clinicians from the National Health Service (NHS) and independent audiology centers agreed to participate in the study. Twenty-nine of the 42 clinicians responded to round 1 (69% response rate), and they formed our expert panel (Fig. 2). Distribution of job roles of the final panel is shown in Table 1. The distribution of the job roles reflects well the UK model of audiologic care for patients with tinnitus, where NHS audiology clinics are the main provider of services for patients with tinnitus. Senior audiologists tend to have more specialist knowledge and more experience than audiologists at a lower grade of employment. Both groups are represented here. In the United Kingdom, independent sector audiologists and hearing aid dispensers rarely specialize in tinnitus, and this perhaps explains why they were less represented in the panel.

**TABLE 1.**
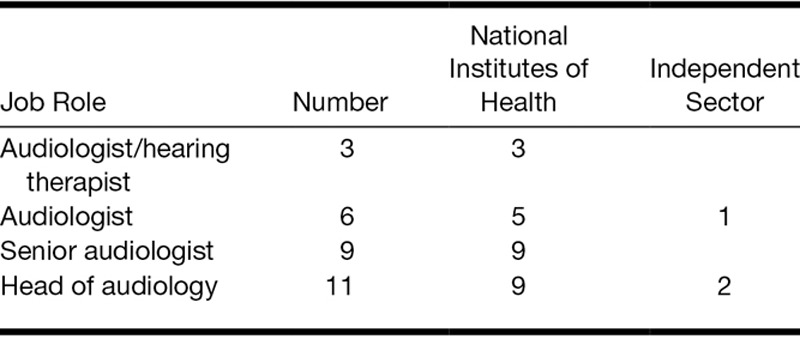
Distribution of job roles of the expert panel

### Stage 3: Design of the “Round 1” Open-Ended Questionnaire

For round 1, an initial set of 10 general open-ended questions was formulated by the study team. Question topics were informed by previous survey results (Hoare et al. 2012), UK Department of Health (2009) Good Practice Guide, National Institute of Clinical Excellence (NICE) report (Taylor & Paisley 2000), and UK professional body guidelines on fitting and verifying hearing aid fit (British Society of Audiology [BSA] and British Academy of Audiology [BAA] 2007; British Academy of Audiology 2009; British Society of Audiology Practice Guidance 2012). Two focus groups involving clinicians from Nottingham Audiology Services were organized. In the first focus group meeting, we received feedback on the 10 open-ended questions we had identified. On the basis of this feedback, we formulated a revised set of 14 open-ended questions. We piloted these questions with our second focus group. The purpose of piloting was to confirm the clarity and conciseness of the questions, as well as the appropriate length of the questionnaire. Questions were then revised based on feedback to form the final “round 1” questionnaire. The 14 questions were grouped into four categories:

i. General factors and practices (two questions)
  We asked panelists to list up to five audiologic and nonaudiologic factors that they consider important when fitting hearing aids for patient with mild hearing loss and to identify differences in practice when fitting hearing aid(s) to a patients who have bothersome tinnitus as a main complaint.
ii. Case studies (seven questions)
  We presented panelists with seven audiograms, which varied in shape and the degree of hearing loss (see figures in Supplemental Digital Content 1, http://links.lww.com/EANDH/A175). We asked “Assuming that the patient is motivated to follow your recommendation, under what circumstances and why would you fit a hearing aid to someone with these hearing profiles?” Panelists were asked to identify up to three reasons they would fit a hearing aid to patients with such an audiogram. They were asked to consider patients with and without bothersome tinnitus separately.
iii. Procedures and protocols (four questions)
  Panelists were asked to identify hearing aid technology (devices/products) offered, prescription formulae, and assessments they performed to verify hearing aid(s) fitting for patients with mild hearing loss with and without tinnitus. We also asked about any departmental limitations or protocols, and what panelists would do differently if there were no constraints on what they could offer patients.
iv. Comments
  Panelists were asked for any additional comments relating to this round of the survey.

### Stage 4: Design of the “Round 2” Closed Questionnaire

To identify themes and to formulate closed questions for round 2, responses to open questions from round 1 were subjected to thematic analysis (Boyatzis 1998). Thematic analysis involves coding and categorizing sections of written or transcribed text based on the themes of the text (Taylor & Bogdan 1984; Boyatzis 1998; Joffe & Yardley 2004). A thematic analysis protocol was based on Braun and Clarke (2006) and Hoare et al. (2012) and involved independent analysis by three researchers (M.S., D.J.H., S.S.). This protocol was predefined at the outset of the study. Stage 1 was a familiarization, or immersion process, where each researcher read and reread all the responses to a question. With that specific question in mind, the next stage was an active reading process where responses that appropriately addressed the question were selected. At the same time, the reader looked for recurring themes or concepts running through the responses. In the next step, initial codes were generated. Codes identify a feature of the individual response that appears relevant to the analyst (Boyatzis 1998) and here referred to the most basic meaningful element of the text. Critically, the chosen segment of each response had to maintain the meaning and context intended by the respondent. Codes that were considered to be equivalent were grouped under “proposed themes.” Analysis to this point was conducted independently by three researchers, after which all three researchers meet to agree “codes” and “proposed themes,” revisiting the full data set to confirm the likeness of codes within a theme and the distinctiveness of codes classified under different themes.

The round 2 questionnaire consisted of 115 statements grouped into four themes derived from the thematic analysis process: (i) general factors affecting the decision to fit hearing aid(s); (ii) protocol-driven factors affecting the decision to fit hearing aid(s); (iii) general practice; and (iv) clinical observations. These themes, based on information provided by the panel, rather than predefined categories created by researchers in round 1, are used later to describe the results.

Each statement in the questionnaire was attached to a 5-point Likert rating scale (1, strongly disagree; 2, disagree; 3, neither agree nor disagree; 4, agree; 5, strongly agree) for panelists to quantify their level of agreement with each statement. For question 1, panelists were also asked to choose the five most important factors from the list of 20 that affect the decision to fit hearing aids and rank them in the order of importance (1 = first most important to 5 = fifth most important).

### Stage 5: Design of the “Round 3” Closed Questionnaire—Interpretation/Consolidation and Final Consensus

The round 3 questionnaire consisted of the same list of closed questions used in round 2. For each item, the summary results from round 2 were reported back to the panelists. Results were anonymized, and the mean level of agreement for each of the closed questions was given. Considering this information, panelists were given the opportunity to revise their personal rating of any statement. A comment box was introduced for 20 statements in which low agreement had been observed in round 2. The comment box invited panelists to explain their round 3 rating. Results from the round 3 questionnaire were taken as the final level of agreement.

## RESULTS

The three-round Delphi survey was conducted between October 2012 and June 2013 (Table 2). One expert withdrew from the study after completing round 1 due to personal reasons, leaving 28 panel members completing the round 2 survey (97% response rate).

**TABLE 2.**
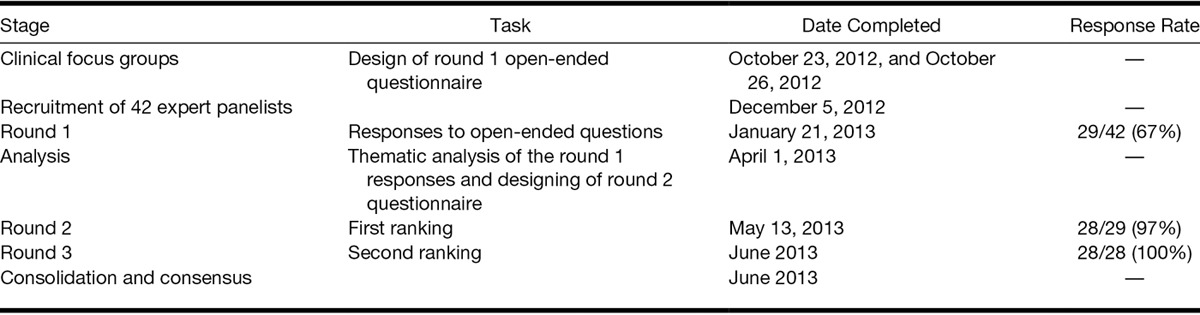
Delphi Study timetable

### Final Consensus and Change in Consensus Between Rounds 2 and 3

Consensus (≥70% agreement) was reached for 58 of 115 statements, with an average agreement of 89.9% (SD = 9.9%) for these statements (Table 3). Kendall’s coefficient of concordance W showed that there was moderate overall agreement between panelists’ ratings for the whole questionnaire (W = 0.59; Siegel & Castellan 1988). When calculated separately for the four themes derived from analysis from round 2 data, agreement between panelist’s ratings varied between 0.49 and 0.56 therefore was in the moderate range (Siegel & Castellan 1988). Kendall’s coefficient of concordance indicated that the agreement between panelists was lowest for theme (iii) “general practice” (0.49) and highest for theme (i) “general factors affecting decision to fit hearing aid(s)” (0.59; Table 4).

**TABLE 3.**
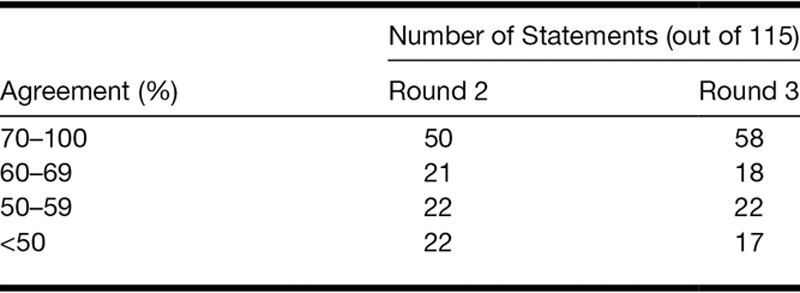
Level of agreement reached by the expert panel

**TABLE 4.**
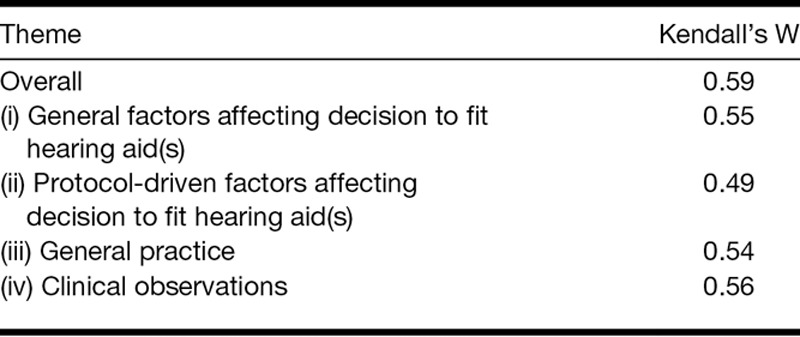
Results of the Kendall’s coefficient of concordance W analysis of final (round 3) agreement between panelists

The number of statements for which the consensus was reached increased from 50 to 58 between rounds 2 and 3. The percentage agreement between rounds 2 and 3 increased for 57 of 115 statements, decreased for 14, and stayed the same for 44 statements. The increase in percentage agreement for 57 statements varied from 1 to 17% (average 6.2%; SD = 4.02), and the decrease for 14 statements varied from 0.5 to 11% (average 3.6%; SD = 3.14). In most cases, the decreases in agreement concerned statements that already had a low level of agreement (<67%) after round 2.

### General Factors Affecting Decision to Fit Hearing Aids

Round 3 identified where there was a panel agreement for the 20 factors that clinicians reported in round 1 to be relevant to the decision about fitting hearing aids for the patient with mild hearing loss but without tinnitus. Greater priority was given by clinicians to patient-centered criteria for fitting hearing aids than the more objective assessment measures.

#### Consensus

Consensus was reached for 11 of 20 factors. Thematic analysis of the panelists’ answers pointed to the importance of two groups of factors when deciding hearing aids candidature for mild hearing loss: patient-centered criteria and objective measures. Patient-centered criteria included patient-reported hearing difficulties, motivation to wear hearing aid(s), realistic expectations, patient self-reported impact of hearing loss on quality of life, general ability to hear in quiet settings and in background noise, and speech discrimination and comprehension in quiet and background noise. Clinicians agreed that among the objective measures, shape of the audiogram, degree of hearing loss, health of the ears, manual dexterity and manipulation difficulties, and hyperacusis/reduced loud sound tolerance all play an important role when deciding whether to fit hearing aids for a mild hearing loss (Table 5).

**TABLE 5.**
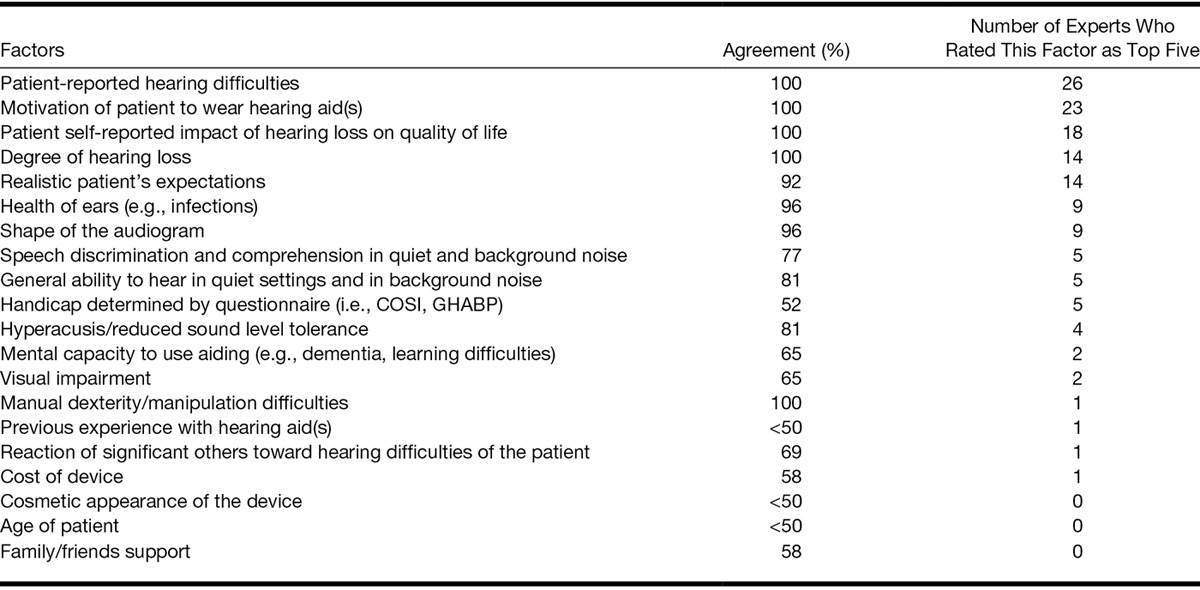
Importance rankings for factors important to consider when fitting hearing aid(s) for mild hearing loss only (without bothersome tinnitus)

#### Importance

Patient-centered criteria were given higher importance than objective measures by our panelists (Table 5). Twenty-six of 28 panelists (93%) listed patient-reported hearing difficulties in the five most important factors, followed by motivation to wear hearing aid(s) (82%), and patient self-reported impact of hearing loss on quality of life (64%). Realistic patient’s expectations and the degree of hearing loss were also listed within the top five important factors by half of the panel (Table 5).

#### No Consensus

Factors for which consensus was not reached are listed in Table 5, together with the percentage of agreement for each. Although they included some subjective factors such as reaction of significant others toward hearing difficulties of a patient and hearing handicap determined by a questionnaire, most factors with a low level of agreement were objective ones including visual impairment, mental capacity to use aiding, previous experience with hearing aid(s), cosmetic appearance of the device, and age.

### Protocol-Driven Factors Affecting Decision to Fit Hearing Aid(s)

In summary, the thematic analysis of round 1 responses identified 14 statements concerning protocol-driven factors affecting the decision to fit hearing aid(s), which formed this part of the survey. While consensus was reached on the opinion that hearing aids are the main intervention for patients with mild hearing loss with tinnitus, there was no consensus on the criteria for determining the candidature both objective (audiometry) and self-reported (questionnaires).

#### Consensus

Consensus was reached for 7 of 14 statements. Panelists agreed that hearing aids should be offered routinely for patients with mild hearing loss and bothersome tinnitus (89%) and that hearing aids are the primary treatment for mild hearing loss with bothersome tinnitus (72%).

On fitting, panel members reached consensus that unilateral hearing aid fitting in patients with mild hearing loss and bothersome tinnitus is not appropriate even if the patient has unilateral tinnitus or an asymmetric hearing loss (96 to 100% agreement depending on the statement, Table 6). Panel members also reached consensus that they would always fit bilateral hearing aids when the patient has a comorbid mild hearing loss and tinnitus (75% agreement), whereas they would offer uni- or bilateral hearing aid(s) for patient without tinnitus depending on the patient’s preference (86% agreement).

**TABLE 6.**
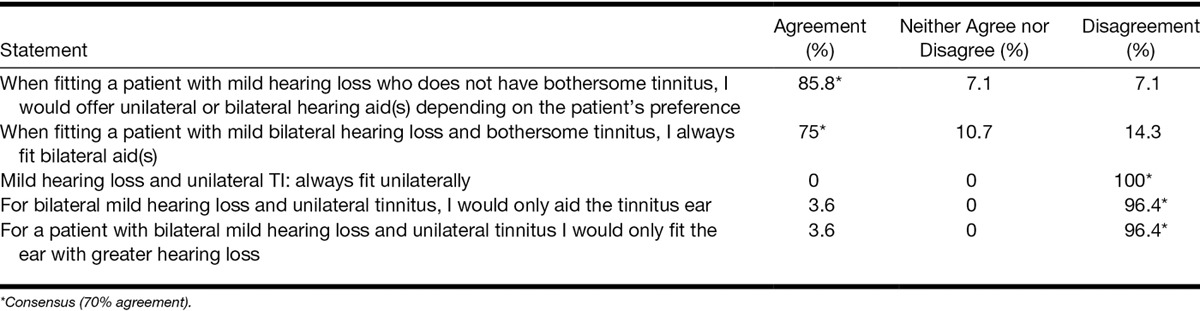
Level of agreement around statements regarding uni- vs. bilateral fit of hearing aids for patients with mild hearing loss with and without bothersome tinnitus

Regarding audiometric criteria for fitting hearing aids for mild hearing loss, panelists reached the consensus that they would offer hearing aid(s) only if patient had a four-frequency pure-tone average worse than 20 dB at least in one ear (75% agreement). They disagreed with the statement that hearing aids should be offered only if the patient showed ≥35 dB hearing loss at 2 kHz (96%; Table 7).

**TABLE 7.**
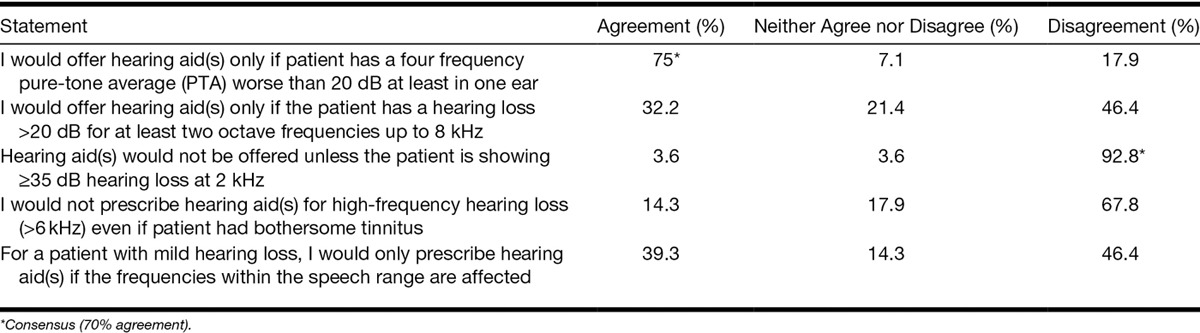
Level of agreement around statements regarding objective audiometric criteria for fitting hearing aids for patients with mild hearing loss with and without bothersome tinnitus

#### No Consensus

Consensus was not reached for statements regarding other potential audiometric criteria for fitting hearing aids (Table 7). These included high-frequency hearing loss (>6 kHz), hearing loss >20 dB for at least two octave frequencies, and affected frequencies being within speech range.

Consensus was not reached for statements on the use of questionnaire scores as a criterion for fitting hearing aid(s) (e.g., tinnitus functional index [TFI, Meikle et al. 2012], tinnitus handicap inventory [THI, Newman et al. 1996] reported by panelists in round 1). Only 68% of the panel agreed that they would base their decision to fit hearing aids on the THI score, and agreement was <50% for the TFI.

### General Practice of Fitting Hearing Aid(s)

In summary, thematic analysis of the round 1 responses identified 48 statements about general “in use” practice for fitting hearing aid(s) for patients with mild hearing loss with and without tinnitus included in this section. Panelists reached consensus for a wide range of statements about candidature for hearing aids, recommended technology, and verification of hearing aids’ fit.

#### Consensus

Consensus was reached for 23 of 48 statements, which we categorized as (i) candidature for hearing aid(s), (ii) hearing aid technology/features, and (iii) verification of hearing aid(s) fit.

i. Candidature for hearing aid(s)
  All panelists agreed (100%) that they would always provide information about the potential benefit of hearing aids for tinnitus management. There was consensus that hearing aid(s) should be offered for patients with a mild hearing loss and bothersome tinnitus even if they did not report hearing difficulties (82%). Panelists also agreed that they were more likely to fit hearing aid(s) for mild hearing loss if the patient also had bothersome tinnitus (86%). Ninety-three percent agreed that they would offer hearing aid(s) to a patient with a mild hearing loss and bothersome tinnitus if the patient did not want or could not use a noise generator.
ii. Hearing aid technology/features
  Consensus was also reached around hearing aid technology/features offered. Generally for mild hearing loss, clinicians would use open fit (100%), behind the ear or on the ear devices (100%) with multiple programs (93%), directional microphone (96%), and slim/thin tubing (93%). For patients who also have bothersome tinnitus, panelists would especially focus on providing as open fit as possible (100%). For someone with tinnitus, they would also aim to reduce feedback (93%), use slim/thin tubing (93%), use separate programs for optimizing speech and audibility (71%), and use frequency compression for high-frequency hearing loss, that is defined as mild loss according to the 4-point (0.5, 1, 2, 4 kHz) pure-tone average (71%).
iii. Verification of hearing aid(s) fit
  Objective (real ear measurements [REMs]) and subjective measures (interview or informal discussion, loud sound check, and live voice/everyday sounds perception) were identified as important to verify the fit (Table 8).

**TABLE 8.**
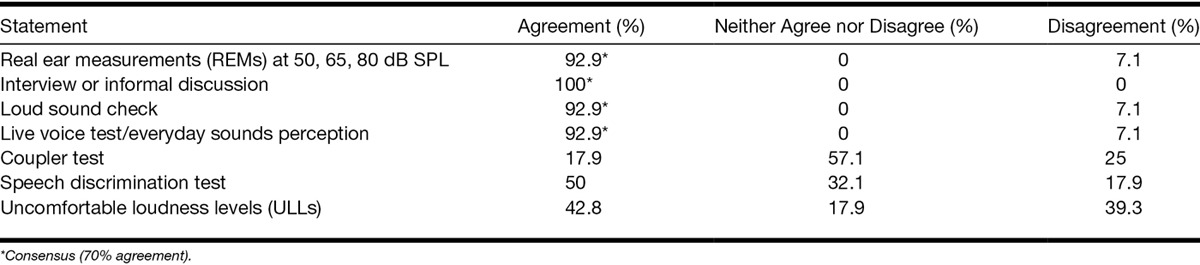
Level of agreement around statements regarding assessments to verify hearing aids fitting for patients with mild hearing loss with and without bothersome tinnitus

#### No Consensus

Consensus was not reached on which formulae to use when fitting hearing aids for patients with and without tinnitus (National Acoustic Laboratories’ nonlinear fitting procedure, version 1 [NAL-NL1] and version 2 [NAL-NL2], Desired Sensation Level [DSL; Seewald et al. 2005], manufacturer’s own, default settings followed by freehand setting based on patient’s reaction; <50 to 68% agreement). There was also no consensus on the safety of performing REMs at 80 dB SPL, uncomfortable loudness levels (ULLs), and loud sound check in patients with tinnitus (<50% agreement). Consensus was not reached on matters concerning whether the coupler test and speech discrimination test were important to verify the fit (<50% agreement; Table 8).

### Clinical Beliefs

In summary, thematic analysis of round 1 responses identified practices that were influenced by personal beliefs and observations totaling 33 statements. Among those were beliefs about the mechanisms by which hearing aids may improve tinnitus and the relationship between hearing aid efficacy and tinnitus pitch.

#### Consensus

Consensus was reached for 17 of 33 statements. Panelists believed that hearing aids improve tinnitus by providing environmental sound enrichment, recalibrating auditory gain/central gain, reducing the concentration or listening effort needed to hear (strain to hear), reducing stress caused by hearing difficulties, and providing distraction from tinnitus (see Table 9 for levels of agreement).

**TABLE 9.**
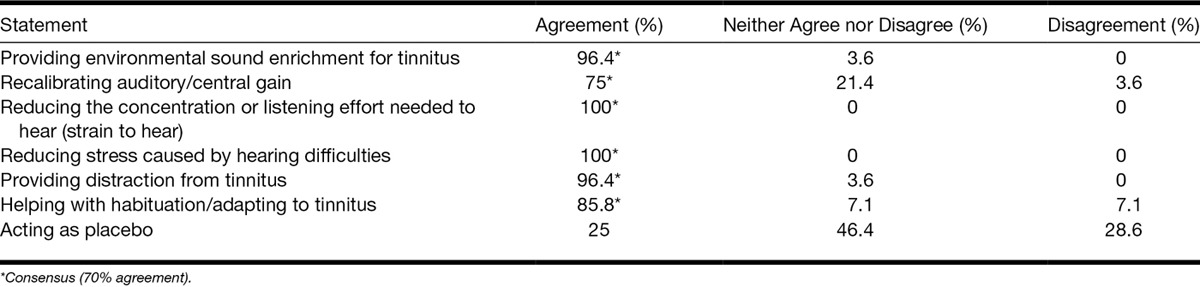
Level of agreement around statements regarding mechanisms by which hearing aids are effective for tinnitus

Seventy-nine percent of panelists agreed that hearing aid(s) reduce tinnitus for patients with mild hearing loss, even if there is no benefit for hearing. However, 75% also believed that hearing aids for low-frequency hearing loss are effective for tinnitus only if the tinnitus is also low frequency and vice versa. All panel members agreed that open-fit hearing aid avoids occlusion, making use of natural hearing, and 75% of members agreed that unilateral aiding can shift tinnitus perception to the unaided ear.

#### No Consensus

Consensus was not reached on whether patients with mild hearing loss are more likely to stop using hearing aid(s) than patients with more significant hearing losses (<50% agreement) and whether patients with a mild hearing loss and bothersome tinnitus are more likely to use their hearing aid(s) compared with patients with a mild hearing losses but without tinnitus (64%). Sixty-one percent of clinicians disagreed with the statement that there was a trade-off between what benefit hearing aid(s) can provide for hearing loss and for tinnitus if patients have both complaints. There was no consensus as to whether questionnaires (Glasgow Hearing Aid Benefit Profile [GHABP], Gatehouse 1999) or Client Oriented Scale of Improvement (COSI, Dillon et al. 1997; <50%) are a good predictor of hearing aids benefit. There was also no consensus that questionnaires are a good outcome measures (THI or TFI; <50%). Some panelists believed that COSI and GHABP questionnaires did not provide much more information than would a conversation with the patient combined with history taking, while some of them thought that questionnaires help to highlight difficulties or are a good starting point for discussion. The panel was not of the consensus that research evidence demonstrates hearing aid efficacy for tinnitus in patients with a mild hearing loss nor that they are beneficial for tinnitus as a placebo (<50%).

## DISCUSSION

This study aimed to identify where there is consensus and lack of consensus amongst experienced clinicians in the United Kingdom on hearing aid candidature and the clinical practice of fitting hearing aids for a mild hearing loss, in patients with and without bothersome tinnitus. Consensus was reached on a range of statements related to what influences the decision making, clinical practice, and common clinical observations when fitting hearing aids for mild hearing loss with and without tinnitus. However, there was also a range of statements for which consensus was not reached. Stability of responses between rounds 2 and 3 was high (mean change = 4.4%, SD = 4%), which indicates some strongly held opinions among hearing professionals. The relatively low number of statements (58 of 115) for which consensus (≥70% agreement) was reached and only moderate agreement between experts, as shown by Kendall’s coefficient of concordance W, reflects the lack of a link between outcomes of the diagnostic assessment and choice of treatment in current practice guidelines (Hoare & Hall 2011; Baguley et al. 2013). Clinicians base their decisions mainly on personal experience and schools of tinnitus practice.

### Professional Guidelines

The lack of high quality research evidence demonstrating effectiveness of certain tinnitus management strategies, as well as lack of linkage between assessment and diagnosis and recommended management strategies, leads to marked differences in clinical practice across different countries. In the United Kingdom and some other regions such as Scandinavia, the Netherlands, and the United States, audiology is an independent profession and the main provider of tinnitus services. However, in other countries such as Germany and Poland, audiology and therefore tinnitus services are a subspecialty of otolaryngology (Baguley et al. 2013). As a result, clinicians take different approaches to tinnitus management depending on their background. For example, neither the UK Good Practice Guide nor the TRI algorithm contains specific guidelines on the provision of hearing aids for patients with hearing loss and tinnitus. Guidelines for the provision of adult tinnitus services each emphasize different aspects of tinnitus management. For example, the Department of Health Good Practice Guide (2009) represents a patient-centered approach reflecting opinions of the multidisciplinary team of authors. In contrast, the TRI algorithm promotes a medical model of tinnitus management.

Depending on the country and treatment pathway, hearing aids may have a different place in the more complex management strategy. Some recent examples in the literature include progressive audiologic tinnitus management (PATM, Henry et al. 2008) in the United States and a stepped-care tinnitus management (Cima et al. 2012) in the Netherlands. In the case of progressive audiologic tinnitus management, patients who require amplification due to hearing loss receive amplification quite early in the process, and authors commented that this can often result in satisfactory management with minimal education and support specific to tinnitus (Henry et al. 2008). However, Cima et al. (2012) concluded that for patients with bothersome tinnitus a stepped-care tinnitus management approach is more effective for improving quality of life and reducing tinnitus severity than is usual care concentrated mainly around audiologic (device-based) interventions.

The most common audiologic management strategy in the United Kingdom is education and reassurance, combined with sound therapy with hearing aids being the most popular choice for patients with tinnitus and a comorbid hearing loss. Where needed, it is supplemented with an intervention to reduce distress such as relaxation therapy or CBT (Baguley et al. 2013). This was reflected in the opinions of the expert panel in the present study. Hearing aids are the primary treatment for a mild hearing loss and tinnitus and should be offered routinely for that group of patients together with information on the potential benefit of hearing aids for tinnitus. However, panelists acknowledged that there are also different treatment options that they would consider at the same time as hearing aids, such as noise generators, CBT, counseling, sound enrichment, or relaxation.

### Hearing Aid Candidature

None of the practice documents referred to earlier specify any objective audiometric criteria for fitting hearing aids for patients with tinnitus. They typically recommend only that hearing difficulties should be addressed, ignoring issues such as tinnitus pitch that may be of importance (McNeill et al. 2012). The term “aidable hearing loss” is often used without including any definition. The only recommendation from the Department of Health Good Practice Guide (2009) that specifies the minimal degree of hearing loss that should be aided is based on the results of a very simple screening test at limited range of frequencies using a hearing screening device. This is a hand-held device that produces a fixed series of six pure tones (75, 55, and 35 dB HL at 3 kHz and 55, 35, and 20 dB HL at 1 kHz). If patients can hear all six tones, the guidelines indicate that they are unlikely to need further hearing assessment and may be discharged after an explanation and advice, even if they have tinnitus. Failing to hear at least one of the tones indicates that patient is likely to have hearing difficulty that would benefit from fitting of hearing aid(s). The device has been piloted in 11 UK primary care practices (Davis et al. 2012). However, there are no data available on its wider use, and it was not one of the criterion listed by panelists in this study.

One important step in tinnitus management concerns determining whether the patient needs audiologic intervention for hearing, for tinnitus, or for both (e.g., progressive audiologic tinnitus management by Henry et al. 2008). Discriminating between distress caused by hearing loss and distress caused by tinnitus seems crucial to plan appropriate interventions. Henry and colleagues proposed different approaches depending on whether reported distress is related mainly to hearing loss or tinnitus and pointed to the fact that patients often confuse the effects of hearing loss with the effects of tinnitus. Henry has proposed that appropriate diagnosis of the difficulties with a questionnaire (e.g., Tinnitus and Hearing Survey, Henry et al. 2008) allows clinicians to determine and address hearing- and tinnitus-specific problems, and only patients showing hearing-related problems, independent of tinnitus, would be offered amplification. This opinion was not shared by panelists in the present study. Our panelists considered the presence of bothersome tinnitus alone, without reported hearing difficulties, a sufficient criterion for fitting hearing aids. Generally, panelists chose patient-centered criteria as important when making a decision about fitting hearing aids, which is in line with the Department of Health Good Practice Guide document (2009) that places more emphasis on holistic, individual care than on standardization (Hoare & Hall 2011). Despite the above, there was a lack of consensus whether self-reported questionnaires for assessing aspects of auditory disability, auditory handicap, and improvements in hearing ability (i.e., GHABP, COSI) are a good predictor of hearing aid benefit for a mild hearing loss. There was also a lack of agreement about the usefulness of self-reported tinnitus handicap questionnaires (i.e., THI, TFI) in predicting hearing aid benefit for tinnitus. Therefore, our panelists did not agree that the decision to fit hearing aids should be based on a questionnaire score. This is surprising as both Department of Health Good Practice Guide (2009) and TRI algorithm (Biesinger et al. 2011) recommend the use of standardized questionnaires such as THI or Goebel-Hiller Tinnitus Questionnaire (Goebel & Hiller 1994). The suggested diagnostic value of tinnitus questionnaires in the TRI document (Biesinger et al. 2011) helps to grade tinnitus severity and identify the urgency of treatment. UK practice would indicate that these matters are assessed through patient interview.

The Guideline for Audiologic Management of the Adult Patient developed by the AAA Task Force (2006) also postulates that care must be patient centered, including creating patient-specific fitting goals. Some of the potential nonauditory predictors of success with amplification listed in the above document are the same as those that our panelists suggested might be important for the decision to fit hearing aids including motivation, realistic patient expectations, manual dexterity, cognition, prior experience with amplification, and visual acuity. However, not all of them achieved our predefined level of consensus.

### Clinical Test Battery

The Department of Health and TRI documents recommend that a diagnostic assessment of a patient with tinnitus should include a case history, audiologic assessment (audiometry, stapedial reflexes, otoacoustic emissions [OAEs]), psychoacoustic tinnitus measures (minimum masking level, loudness, pitch), and assessment of tinnitus severity and psychiatric comorbidity (depression, anxiety) using standardized questionnaires (Department of Health 2009; Biesinger et al. 2011). While the degree of hearing loss and the shape of the audiogram (audiometry) were factors important to consider when fitting hearing aids, reflexes and OAEs do not seem to be a standard choice among our panelists. This is in line with our previous report, where 91% of clinicians reportedly conduct audiologic examination routinely, while only 2% reported routinely conducting OAEs and stapedial reflexes (Hoare et al. 2012). Stapedial reflexes can provide information about the type and degree of hearing loss (Gelfand 2009), while the results of OAEs test is accepted as the evidence of outer hair cells functioning (Brownell 1990). Some hypotheses link outer hair cell dysfunction to tinnitus generation (Lonsbury-Martin & Martin 2004). There is no clear evidence of the diagnostic value of OAEs testing for tinnitus, but the results of the OAEs test could be useful for counseling and educational purposes to assist the patient in understanding outer hair cells as a potential site of tinnitus generation (Henry 2004). Psychoacoustic measures of tinnitus were not identified here. This is in line with the result of the survey by Hoare et al. (2012) as only 17% of clinicians reported that they use psychoacoustic measures to assess tinnitus. The value of obtaining tinnitus psychoacoustic measures is commonly questioned, and the results of these tests are not generally perceived useful for diagnostic or therapeutic decision making (Henry 2004; Tunkel et al. 2014).

Both the Department of Health and TRI also recommend the use of a tinnitus questionnaire to measure tinnitus severity. Newman and Sandridge (2004) postulated that tinnitus questionnaires are instrumental in determining candidacy for treatment and choosing the right management approach, especially given there is often a mismatch between the psychoacoustic characteristics of tinnitus percept and the self-perceived disability. They suggested that while a single counseling session might be sufficient for a patient with a low score on the THI questionnaire, a patient with a high score might require intensive and more complex management. Despite the above, there was no agreement on the use of questionnaires for assessing tinnitus severity among panelists. The THI is the most commonly used, with some panelists mentioning the TFI as an alternative. The TFI is however a relatively new measure, and our panelists commented that they are not yet familiar with it. Those who were familiar with TFI expressed the opinion that it seems to be more specific and a more sensitive measure than the THI. The advantages of using THI suggested by the expert panel were that it is quick to administer and gives a general idea of how the patient is managing with their tinnitus. The main disadvantage seemed to be that it gives only very general idea about perceived handicap and does not discriminate between specific domains (e.g., when someone is troubled with poor sleep). The TFI, on the contrary, has been designed to cover multiple tinnitus severity domains panelists noted that it has not been validated in the United Kingdom; therefore, one cannot be sure if it is appropriate for the UK population. Other comments highlighted that the TFI only considers tinnitus over the past week, which could influence the results if the patient’s tinnitus fluctuates.

It seems that despite guidelines regarding diagnostic assessments for patients with tinnitus being relatively consistent among documents (Department of Health 2009; Biesinger et al. 2011), most clinicians perform only part of recommended battery of tests. Lack of adherence to any standardized assessment battery also makes it difficult to compare different management strategies and identify best practices.

### Fitting Hearing Aids

Standard fitting formulae such as NAL-NL1 and more recently NAL-NL2 are the preferred formulae in NHS audiology departments (British Society of Audiology and British Academy of Audiology Guidance 2007). However, there was no consensus on which of the two is better for fitting hearing aids for a mild hearing loss and if one has an advantage over the other when the patient also has bothersome tinnitus. One reason might be that NAL-NL2 is a relatively new formula (Keidser et al. 2011). Comments indicated that not all panelists had experience using NAL-NL2.

Recent work by Shekhawat et al. (2013) concluded that DSL might be a good starting point for the prescription of hearing aid output for tinnitus management, particularly for patients with lower tinnitus pitch. For these patients, DSL prescribes more low intensity and more low-frequency gain than NAL-NL1 therefore potentially provides greater masking. A similar result was also found in an earlier study by Wise (2003), where tinnitus was less audible when hearing aids were programmed using DSL rather than NAL-NL1 formulae, while participants preferred NAL-NL1 for speech perception. Wise (2003) recommended two separate programs, one optimized for speech intelligibility and another for tinnitus relief.

Consensus among panelists in our study that NAL-NL1 puts emphasis on speech intelligibility and high-frequency gain needed for speech clarity is in line with the evidence provided by the above studies, as was consensus to offer separate programs for tinnitus patients: (1) with settings to optimize speech comprehension and (2) with settings to increase the audibility of background sounds. However, despite the above evidence, panelists would use the DSL formula mainly for children or patients who had previous experience with DSL. There was no consensus on whether the upset caused by tinnitus or hearing difficulty was more important to address when fitting hearing aid(s). Comments indicated that our panelists try to address both issues, but they are aware of a possible trade-off between fitting decisions that are optimized for tinnitus benefit and those optimized for listening satisfaction.

### Unilateral Versus Bilateral Fitting of Hearing Aids

Another interesting question is whether a patient with tinnitus should be offered unilateral or bilateral hearing aids. Although panelists agreed that unilateral fitting is not appropriate for patients with tinnitus, this conviction is not well evidence based.

Some authors report an improvement in tinnitus handicap with both unilateral and bilateral hearing aids regardless of the laterality of tinnitus (Brooks & Bulmer 1981; Tyler & Bentler 1987; Trotter & Donaldson 2008). Other authors postulated that in the case of a unilateral hearing loss and tinnitus fitting the impaired ear is sufficient, whereas individuals with bilateral complaints require bilateral fitting (Melin et al. 1987; Zagólski 2006). However, the efficacy of hearing aid fitting for tinnitus rather than the laterality of the fitting was the primary question and none of the studies to date offer high quality evidence for or against (Hoare et al. 2014).

Here, the panelists indicated that for patients who do not have a bothersome tinnitus they would base their decision to offer unilateral or bilateral aid(s) on patient preference. However, in the case of a bothersome tinnitus, the consensus was that unilateral fitting of a hearing aid in patients with a bothersome tinnitus is not appropriate even if the patient has a unilateral tinnitus or an asymmetric hearing loss. Comments indicated that clinical observations suggest that unilateral fitting of hearing aids in patients with tinnitus may “shift” the tinnitus sensation to the unaided ear consistent with the observation by Zagólski (2006). However, a mechanism to explain this phenomenon is not clear. One explanation might be that patients for whom the perception of tinnitus shifts to the unaided ear have a bilateral tinnitus that is louder in one ear, therefore creating a unilateral perception. In such cases when fitted with one hearing aid, patients’ tinnitus might improve on that side making the tinnitus in the other ear more noticeable (Blauert 1997). Another possibility is that unilateral aiding compensates for a decrease in central gain on one side only (Schaette et al. 2012).

### Assessments to Verify Hearing Aid Fit

Differences in practice in the management of mild hearing loss with and without tinnitus are also seen in the choice of assessments that clinicians considered important to perform to verify hearing aid(s) fitting. While consensus was reached for most measures, there was no agreement on the safety of performing REMs at the highest loudness level (80 dB SPL), testing ULLs, and loud sound check for patients with tinnitus. Some panelists commented that performing these assessments could exacerbate tinnitus. The BSA-recommended procedure for the determination of ULLs states that they are generally helpful for hearing aid fitting, although most hearing aid fitting software contains normative ULL values that may be used instead (British Society of Audiology 2011). At the same time, the document states that ULL testing should only be performed when the audiologic professional considers the information to be clinically useful, leaving the choice to the clinician. The BSA-recommended procedure states that the main contraindication to perform this test is a “significant” tinnitus, which can sometimes be exacerbated by ULL testing. This may explain variability in clinical practice.

### Hearing Aid Technology

The presence of tinnitus did not influence the choice of technology/hearing aid features for a mild hearing loss. In line with the Department of Health guidelines (2007), open ear tip technology that avoids occlusion and makes use of residual hearing would be used for a mild hearing loss with and without tinnitus (Del Bo et al. 2006).

## CONCLUSIONS

This study reports on the consensus for hearing aid candidature and fitting in cases of mild hearing loss with and without bothersome tinnitus. Hearing professionals generally support the use of such technologies especially when the patient reports hearing difficulties and has realistic expectations of the technology and a bothersome tinnitus. Practices such as the use of open-fit technology and bilateral fitting of hearing aids for patients with bothersome tinnitus would seem to constitute “usual care.” Common limitations of guideline documents for diagnosis and management of tinnitus are the lack of a clear link between the outcomes of the diagnostic test battery and the recommended treatment, as well as guidelines being too general and lacking reference to the appropriate assessment tools (i.e., recommended questionnaires), which leads to variability in clinical practice.

## ACKNOWLEDGMENTS

We thank members of the Delphi expert panel for participating in the study and clinicians form Nottingham Audiology Services for their help in developing questionnaires used in this study. We also thank Vicky Owen for providing statistical support.

## Supplementary Material
