# Supplementary material for: Consensus on Hearing Aid Candidature and Fitting for Mild Hearing Loss, With and Without Tinnitus: Delphi Review
Source: Ear Hear. 2015 Jun 24;36(4):417–29. doi: 10.1097/AUD.0000000000000140 (PMC4478070; doi:10.1097/AUD.0000000000000140)
Supplement: Supplementary file 1 [file aud-36-0417-s001.docx]

Supplemental Digital Content 1: Audiograms presented to the clinicians in the Round 1 questionnaire. The question asked was: *Assuming that the patient is motivated to follow your recommendation, under what circumstances and why would you fit a hearing aid to someone with these hearing profiles? Please consider both patient examples (A and B) and provide up to 3 circumstances for each patient.*

|  | Patient A: without a bothersome tinnitus  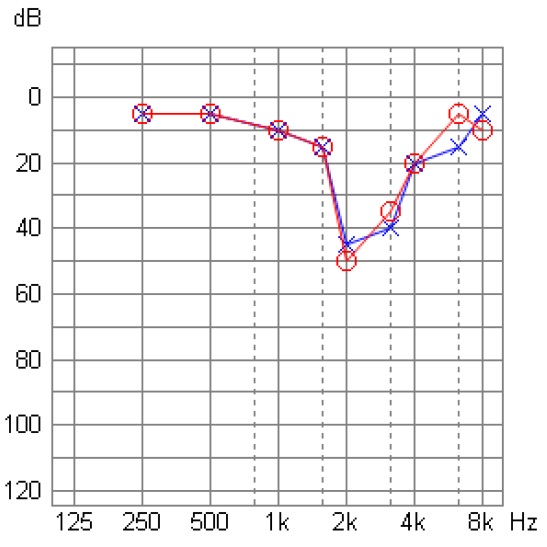  PTA (4 pt)  R=21  L=20 |  | Patient B: with a bothersome tinnitus  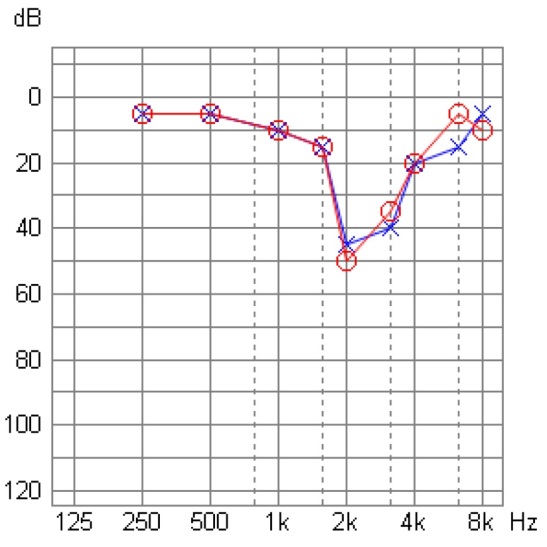  PTA (4 pt)  R=21  L=20 |
| --- | --- | --- | --- |

|  | Patient A: without a bothersome tinnitus  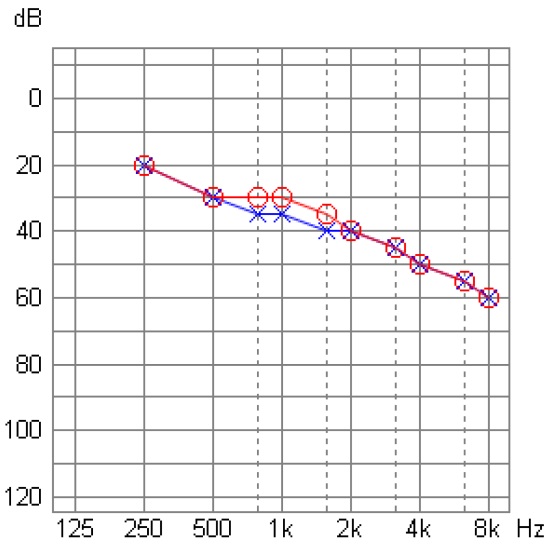  PTA (4 pt)  R=37  L=39 |  | Patient B: with a bothersome tinnitus  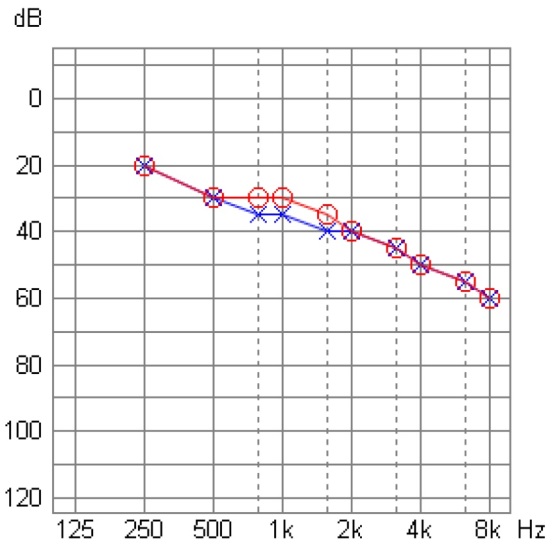  PTA (4 pt)  R=37  L=39 |
| --- | --- | --- | --- |

|  | Patient A: without a bothersome tinnitus  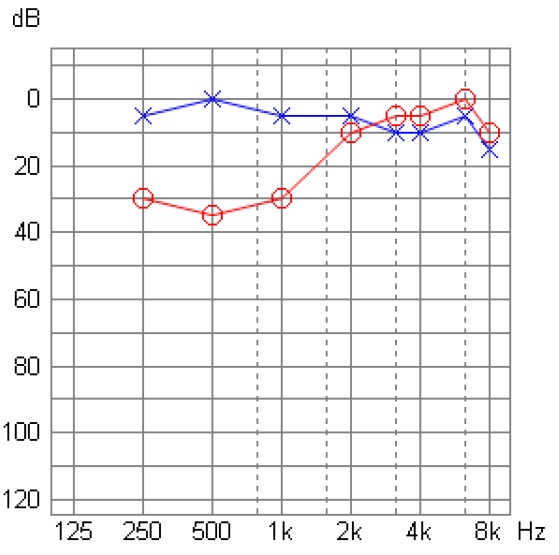  PTA (4 pt)  R=20  L=5 |  | Patient B: with a bothersome tinnitus  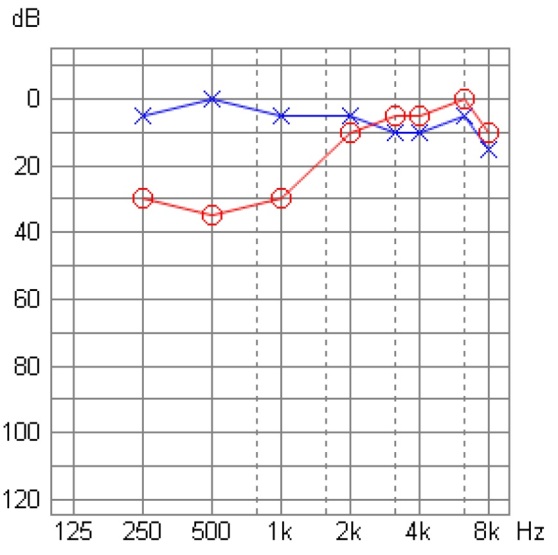  PTA (4 pt)  R=20  L=5 |
| --- | --- | --- | --- |

|  | Patient A: without a bothersome tinnitus  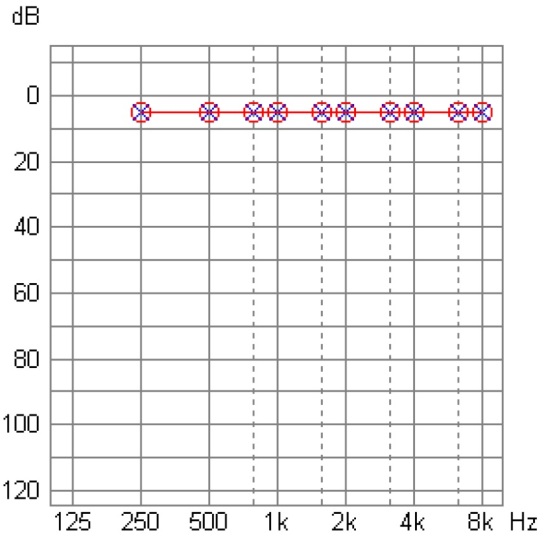  PTA (4 pt)  R=5  L=5 |  | Patient B: with a bothersome tinnitus  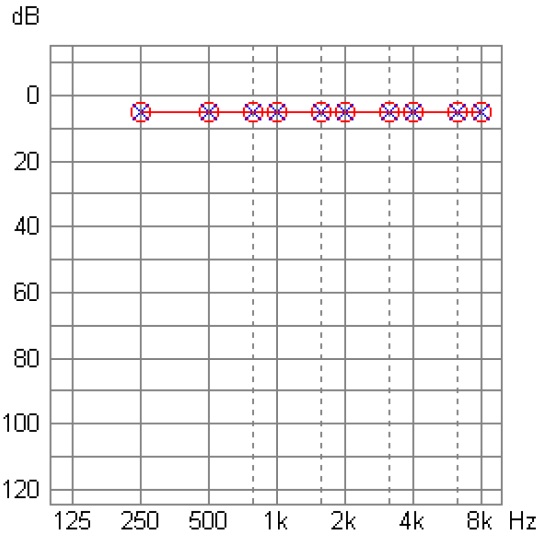  PTA (4 pt)  R=5  L=5 |
| --- | --- | --- | --- |

|  | Patient A: without a bothersome tinnitus  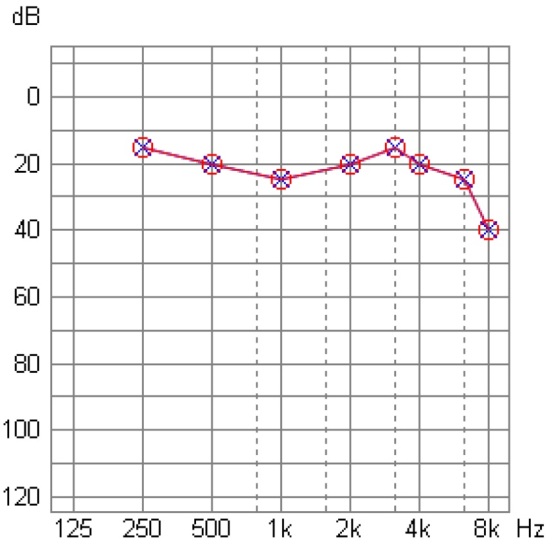  PTA (4 pt)  R=21  L=21 |  | Patient B: with a bothersome tinnitus  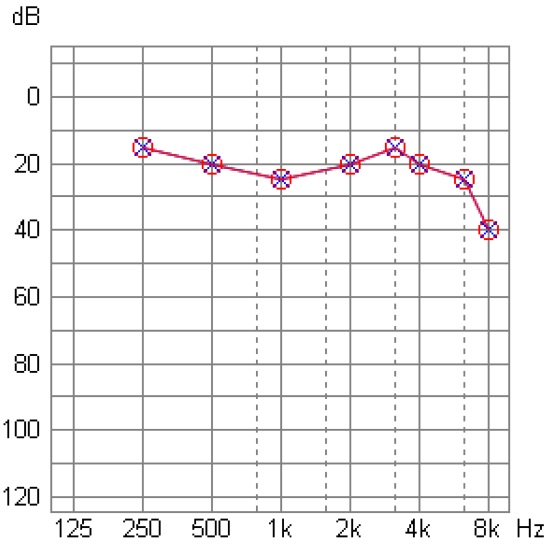  PTA (4 pt)  R=21  L=21 |
| --- | --- | --- | --- |

|  | Patient A: without a bothersome  tinnitus  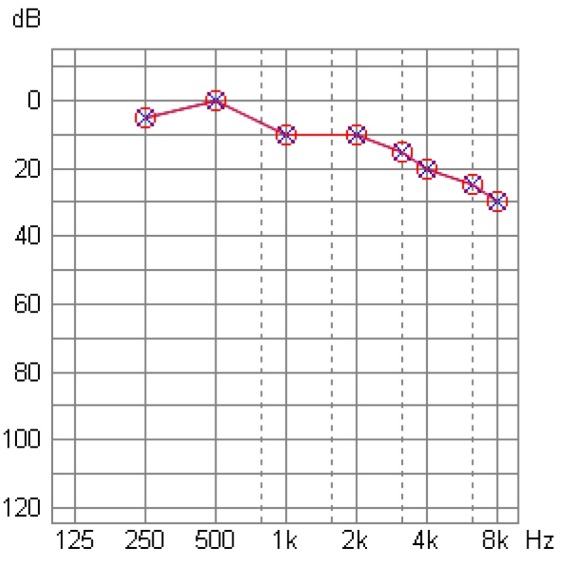  PTA (4 pt)  R=10  L=10 | |  | Patient B: with a bothersome tinnitus  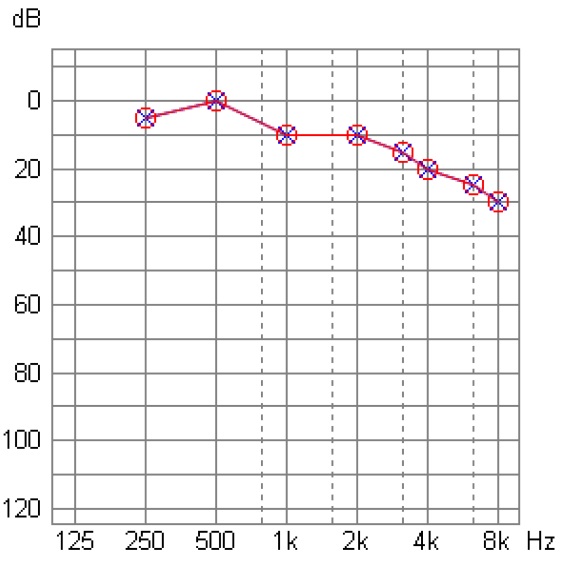  Case studies  PTA (4 pt)  R=10  L=10 | |
| --- | --- | --- | --- | --- | --- |
|  | Patient A: without a bothersome tinnitus  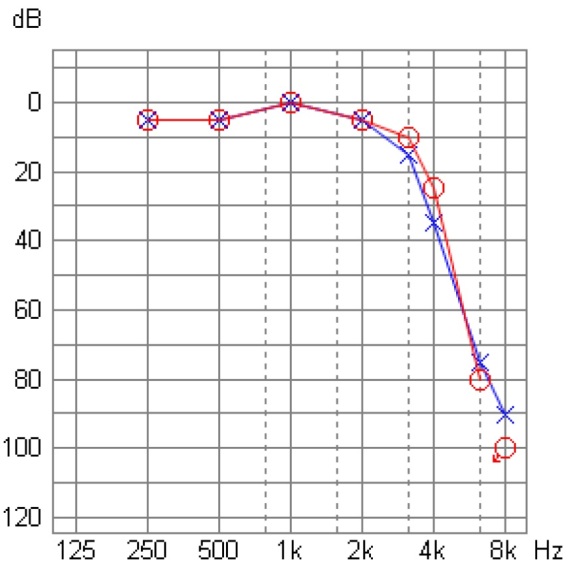  PTA (4 pt)  R=9  L=11 |  | Patient B: with a bothersome tinnitus  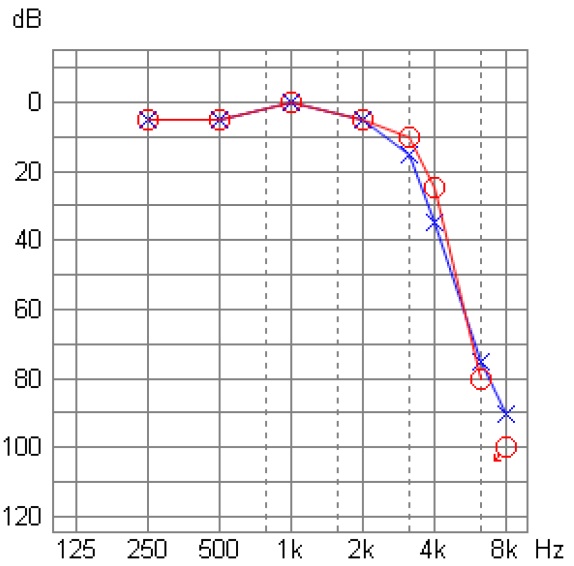  PTA (4 pt)  R=9  L=11 | |  |
